# Introducing DynaPTI–constructing a dynamic patent technology indicator using text mining and machine learning

**DOI:** 10.3389/frai.2023.1136846

**Published:** 2023-05-03

**Authors:** Michael Freunek, Matthias Niggli

**Affiliations:** ^1^EconSight AG, Basel, Switzerland; ^2^Center for International Economics and Business, University of Basel, Basel, Switzerland

**Keywords:** patents, patent intelligence, machine learning, text mining, ESG, green transition

## Abstract

Patent data is an established source of information for both scientific research and corporate intelligence. Yet, most patent-based technology indicators fail to consider firm-level dynamics regarding their technological quality and technological activity. Accordingly, these indicators are unlikely to deliver an unbiased view on the current state of firm-level innovation and are thus incomplete tools for researchers and corporate intelligence practitioners. In this paper, we develop DynaPTI, an indicator that tackles this particular shortcoming of existing patent-based measures. Our proposed framework extends the literature by incorporating a dynamic component and is built upon an index-based comparison of firms. Furthermore, we use machine-learning techniques to enrich our indicator with textual information from patent texts. Together, these features allow our proposed framework to provide precise and up-to-date assessments about firm-level innovation activities. To present an exemplary implementation of the framework, we provide an empirical application to companies from the wind energy sector and compare our results to alternatives. Our corresponding findings suggest that our approach can generate valuable insights that are complementary to existing approaches, particularly regarding the identification of recently emerging, innovation-overperformers in a particular technological field.

## Introduction

Heat waves, droughts or melting glaciers in different parts of the world underline the need for a rapid green transition to mitigate the implications and to approach the challenges of climate change. While this involves structural changes across all sectors of today's society, the invention of new green technologies and their adoption by businesses play a key role for achieving a sustainable green transformation (see e.g., Davis et al., 2018; Sachs et al., 2019). At the same time, a green transition requires the mobilization of vast investments and the development of new green inventions (see e.g., Fagerberg, 2018; Polzin and Sanders, 2020), which is one reason why the so-called environmental, social and governance (ESG) framework has become an important tool to facilitate funding for the necessary technological transitions.^1^ However, even though there have been extensive efforts to provide transparent and objective ESG ratings for organizations, problems such as greenwashing persist (see e.g., Laufer, 2003) and ratings can remain imperfect measures for assessing the actual green technological potential of companies (e.g., Cohen et al., 2020).

An alternative approach for investigating companies' technological strengths and green innovation potentials is to leverage patent data.^2^ Due to constantly evolving empirical techniques, patents have become a well-established data source for approximating the innovativeness of companies in scientific research and corporate intelligence (e.g., Acs et al., 2002). This article builds on an extensive literature concerned with the development of empirical approaches to depict the “importance,” the “value” or the “quality” of patents to derive such innovation indicators. An early example is Trajtenberg (1990), who deviates from simple patent counts toward more sophisticated indicators based on patent citation weights. Other studies have proposed additional patent-based metrics, such as the number of claims a patent seeks protection for (e.g., Tong and Frame, 1994), or the geographical scope of a patent's protection (e.g., van Pottelsberghe and van Zeebroeck, 2008). Some subsequent contributions have started to merge different measures into one aggregate indicator which then provides a more comprehensive perspective on quality (e.g., Lanjouw and Schankerman, 2004 or Ernst and Omland, 2011). More recently, researchers have also started to successfully leverage the textual information contained in patent documents to extract quality signals (e.g., Chung and Sohn, 2020; Arts et al., 2021).

However, a large part of the literature has focused on creating *static* measures when ultimately aggregating patent value scores from patents to firm-level indicators (e.g., Ernst and Omland, 2011). The obvious downside of such approaches is that they fail to account for differing innovation dynamics across firms. For example, say a company A has developed 10 important patents 10 years ago. Then this company A would be similarly rated by static patent measures as company B, which has developed 10 equally important patents in just the last year.

In this paper, our aim is to build a framework that builds on the preceding literature but tackles such shortcomings and allows to effectively leverage the fine-grained information codified in patents to construct a *dynamic* indicator that depicts technological strengths and innovative potentials of companies. Different to prior studies in the field, we propose an approach that is based on an index-based comparison of firms and considers how dynamic their inventive activities have emerged over a specific time window. To capture the latter, we build on established concepts from the literature. First, we assess the quality of firms' patents based on patent citations (e.g., Trajtenberg, 1990 or Ernst and Omland, 2011).^3^ Second, we focus on firms' patenting activities over a specific time window based on simple patent counts. While the former provides a measure of how technologically relevant a firm's inventions are to other inventors, the latter indicates how present a particular firm is in a certain technology field. In addition to these indexing and dynamic components of our proposed measure, we also follow insights from recent studies in the field and additionally incorporate machine-learning techniques to enrich our approach based on textual information from patents (e.g., Lee et al., 2018; Chung and Sohn, 2020). We present an illustrative example showing that our developed indicator provides relevant information for assessing firms' technological strengths and innovation potentials. We demonstrate this based on an application of our indicator to firms active in the technology field of wind energy, whereas we perform a comparison exercise to an established patent indicator, as well as to firms' weighting scheme in a popular wind energy exchange traded fund (ETF).

What are potential implications from implementing our framework? Based on our exemplary results, we argue that our indicator may be particularly well suited to identifying companies that are technologically strong in the ESG area. Furthermore, it could be a useful tool to detect firms that engage in greenwashing activities. A final advantage of our framework refers to its flexibility. Although our empirical application focuses exclusively on firms that are currently active in the technology field of wind energy, our methodological framework can be easily adapted and deployed for other use-cases in any other technological domain, such as artificial intelligence or semiconductor technologies. This makes our proposed method a potentially attractive tool for various applications, and we expect it to add valuable additional perspectives to already established patent intelligence measures.

Given its scope, our paper lies at the intersection of different literatures. Firstly, it has a clear connection to contributions that develop indicators based on patent data for economic research or corporate intelligence.^4^ In particular, our paper is closely related to contributions such as Grimaldi et al. (2015), Ernst and Omland (2011) or Allison et al. (2003) who focus on patent-based indicators at the company-level. Our proposed indicator primarily differs from these existing alternatives by introducing a dynamic component and by additionally leveraging textual information from patents. Secondly, as we use our proposed indicator to assess companies that develop green innovations, our analysis is also loosely related to recent research focusing on the effectiveness of the ESG framework (see e.g., Broadstock et al., 2020; Berg et al., 2022) or greenwashing problems (see e.g., Yu et al., 2020; Li et al., 2022). Although we do not focus on these issues directly, our developed framework may provide an additional, empirical perspective to such studies.

The remainder of the paper is structured as follows. Section Patent analytics and patent indicators–a brief overview provides a brief overview of prior work in the field of patent analytics with a focus on the development of patent technology indicators. In Section Introducing DynaPTI-a dynamic patent technology indicator we explain the building blocks of our proposed framework. Section An application to the wind energy sector presents illustrative results from applying our approach to companies from the wind energy sector. Section Discussion and conclusion discusses the main results, gives an outlook, and concludes the paper.

## Patent analytics and patent indicators–a brief overview

Over the last decades, patent data has become one of the most widely used information sources for researchers and practitioners in various areas such as innovation economics, network analyses, competition policy, as well as finance and corporate intelligence. However, patent statistics come in a relatively raw and unstructured form. Hence, a particular challenge is to construct measures that accurately describe companies' inventive activities and their technological strengths. Early indicators have focused on simple counts of patent grants or patent applications at the company level for this task (e.g., Basberg, 1987). Yet, such measures were not able to capture the importance or quality of patents' underlying inventions, and more sophisticated indicators have since emerged. Typically, corresponding methods leverage information from patent citations to approximate technological quality.^5^ The common idea of these approaches are that a patent's number of received citations from other, subsequent patents (so-called *forward citations*) provides an indication of the patent's technological importance, since these citations depict the extent to which other patents are making use and build upon its underlying invention. Notwithstanding this very appealing feature of patent citations, they are also subject to several distorting factors. For example, the citation intensity of patents varies over time and across technological fields and patent offices (see e.g., Graham and Vishnubhakat, 2013; Kuhn et al., 2020). Researchers have thus developed refined approaches to mitigate and correct such citation distortions (see e.g., Jaffe and de Rassenfosse, 2019 for an overview). Additionally, citation-based indicators were often augmented with further information codified in patents, such as a patent's geographical scope of protection, its technological proximity to existing patents measured by backward citations, or the relatedness to the scientific literature (see e.g., Nagaoka et al., 2010 for an overview). In recent years, a growing literature has also started to focus on patents' texts to assess the novelty and disruptive potential of their corresponding inventions (see e.g., Arts et al., 2021; Kelly et al., 2021; Hain et al., 2022).

While several studies making use of patent statistics primarily investigate technological trends or the emergence of new technologies (e.g., Érdi et al., 2013; Kelly et al., 2021), a particular strand of the literature, which our paper is most closely related to, focuses specifically on the innovativeness of companies, regional innovation clusters or even countries.^6^ To do so, patent-level indicators are typically aggregated to so-called patent portfolios (e.g., Grimaldi et al., 2015). For example, patent counts can be summed up to an aggregate regional patent stock, or patents' citations can be averaged over an entire company's patents to obtain more aggregate metrics. However, this may also distort indicators due to size effects or when initial patent portfolio levels are insufficiently considered. Furthermore, even if such distortions are appropriately addressed, portfolio-level indicators generally remain static and only provide information about an entity's innovative potential at a given point in time. This is because corresponding approaches typically fail to account for the dynamics and changing directions of recent patenting activities. Our proposed patent-based technology indicator aims to mitigate such limitations. In the following section, we introduce its building blocks and carefully describe how it includes a dynamic perspective and alleviates potentially distorting factors related to the patent examination process.

## Introducing DynaPTI-a dynamic patent technology indicator

In this section, we formally derive the framework of our proposed *Dynamic Patent Technology Indicator* (DynaPTI), which allows to investigate firms' technological strength and innovative quality using a dynamic perspective. This dynamic perspective is the core of our proposed framework, as it thereby extends the prior literature that typically uses static indicators (e.g., Ernst and Omland, 2011).

Before we start with these formal derivations, let us briefly clarify some subsequently used terminology. Starting with patents, we use the term “patent” to refer to the whole *patent family* as unit of analysis.^7^ Next, when we refer to “patent quality” without specifying it in more detail, this is intentional as any definition of patent quality can, in principle, be used here. With the term “quality” we simply mean any form of evaluation measures at the level of patent families such as the frequency of forward citation. Finally, we use the terms company, firm, owner, and growth, change rate and dynamics interchangeably, even if they are not always to be understood as synonymous outside of this paper.

DynaPTI is an aggregate indicator at the company-level based on the sum of four sub-measures denoted by 1a, 1b, 2a, and 2b. These sub-measures capture the quality and activity dynamics over a given period (1a and 1b) and additionally represent their corresponding average values (2a and 2b). Moreover, the sub-measures are characterized by the following two properties:

First, the four sub-measures are all index-based, which means that their calculation steps take the patents of other companies from the same index as a benchmark into account. This allows to automatically mitigate distorting factors such as a company's size, geographical location, or technological area (see e.g., Nagaoka et al., 2010 for an overview). Furthermore, it allows to sum up the four sub-measures to a single indicator. Second, we use machine-learning techniques to leverage textual information from patents, which allows to approximate the technological quality of firms' innovations, as well as to correct for distorting factors related to the patent examination process. In the following, we provide the motivation and more details about the four sub-measure forming the DynaPTI:

### Sub-measure 1a: dynamic technological quality

A measure that depicts the relative growth of a firm's patent portfolio quality, related to all firms forming an index α. This measure describes, how the quality of the patent portfolios changed over the specified period. The underlying idea to incorporate such a measure is to give preference to companies with a positive quality development over companies in the same index with a deteriorating quality. This particular feature is typically absent in alternative frameworks (see e.g., Ernst, 2017 or Thoma, 2014; Guderian, 2019). The question then becomes how to measure patent quality. In principle, any methodology for deriving the quality of patent portfolios can be used, such as the widespread quality indicators based on patent citation frequencies (see e.g., Hall et al., 2005 or Harhoff et al., 1999; Ernst and Omland, 2011). We generally follow this approach and also use patent citations to measuring patent quality. But additionally extend it by using machine learning techniques to identify a patent's most technologically related patents based on their textual similarities.^8^ This procedure enables to approximate an individual patent's quality based on the quality of its technologically most similar patents. This allows to take into account that the examination process of an individual patent is subject to several distorting factors.^9^

### Sub-measure 1b: dynamic technological activity

A measure that depicts the relative growth of a firm's patent activity, related to all firms forming an index α. Here we understand the patent activity of a company as the annual number of patent publications, which are assigned to the index. Relative growth describes how patent activity changes over time. This step is intended to give preference to companies with increasing compared to companies with decreasing patent activity. This sub-measure is included following insights from recent research that highlights that there is a correlation between patenting activity and innovative originality (Bedford et al., 2021).

In addition to the relative dynamics of patent quality and activity, we also consider the absolute quality and activity of patent portfolios with the following two measures:

### Sub-measure 2a: average technological quality

A measure that depicts the mean value of a firm's patent portfolio quality, related to all firms forming an index α. This step is intended to give preference to companies with a higher patent portfolio quality over companies in the same index with a lower quality. Thereby, this measure controls for the fact that a qualitatively lower ranked company's patent portfolio can be more easily improved compared to an already highly ranked competitor. Additionally, companies with a higher quality of the patent portfolio are associated with a higher innovative power (Ernst and Omland, 2011; Thoma, 2014; Ernst, 2017; Guderian, 2019).

### Sub-measure 2b: average technological activity

A measure that depicts the mean value of a firm's patent activity related to all firms forming an index α. This step is intended to give preference to companies with high patent activity compared to companies with a low patent activity. The rationale to incorporate this sub-measure follows to one outlined in 2a, which is to correct for potential bias in favor of smaller companies in the 1b measure.

Before we formally state the detailed calculation of these four sub-measures, we next show how we define an index and how we use machine learning to textually enhance the patent quality estimation.

### Index definition

To derive the index-based metrics, we consider a set of patents denoted by *M*_α*, i*_, *M*_α*, i-*1_, *M*_α*, i-*2_… *M*_α*, i-n*_, which are assigned to an index α and are published during the time periods represented *by i, i-*1*, i-*2 …*i-n*. These patents *M*_α*, i*_, *M*_α*, i-*1_, *M*_α*, i-*2_… *M*_α*, i-n*_ of index α assigned to a firm *f* are given by *M*__*f*_, α*, i*_, *M*__*f*_, α*, i-*1_, *M*__*f*_, α*, i-*2_… *M*__*f*_, α*, i-n*_ and are defining the patent portfolio published by firm *f* assigned to the index α. The index α consists of *c* firms, i.e., the number of firms forming the index α is *c*. Examples of such indices may be (1) the firms forming a stock index, e.g., the Nasdaq-100 for US firms, where *c* =100, (2) the firms assigned to a specific technology field, (3) the firms headquartered in a selected country, (4) the firms listed in a fund or firms active in an ESG technology.^10^ The due date *i* represents end of year *i*, e.g., if *i* refers to 2022, it represents the end of the year 2022, *i*-1 then represents the end of the year 2021 etc. This means that the duration from due time (*i–x*) *to* (*i-x* + *n*) is *n* years. At due time *i*, only active patent publications are accounted for, and the same holds true for *i-*1*, i-*2, and so on.

This definitions and assignment processes allow all subsequent investigations to be index-specific.

### Using machine learning to approximate technological quality

Although several approaches are possible to depict a patent's technological quality, it is typically approximated by a patent's citations it received from patent offices. Our framework follows this approach but can also be flexibly adapted to other quality definitions. Hence, the following describes our concept to approximate patents' technological quality in very general terms, so it can be identically applied to any other kind of quality measure at the patent level.

As a starting point, recall that existing patent quality indicators typically leverage information from patent citations. The idea behind this approach is simple: When an arbitrary patent A (that we want to assess according to its quality) is cited by a subsequently applied patent B filed at some patent office, its disclosed technology is somehow related to patent B. Often patent A is relevant to novelty for one or more claims in patent B which define the scope of the invention in patent B.

However, using patent A's raw number of received citations as a proxy for its technological quality can be problematic, since received citations can vary greatly due to several reasons. On the one hand, the patent office's examiner may have made a misjudgment, or the quoted text passages from patent A do not relate to the core of the invention of patent A. In addition, examiners may be biased and prefer to cite patents from their own geographic area and possibly from specific companies. It is also conceivable that once cited patents appear in later citation analyses, they are cited more frequently than patents that have not yet been cited, but which describe the same or at least very similar technical content. Patent A can also be too young to appear in a citation, although it is technologically located in a highly competitive and highly valued technology-environment.

This is where the idea of a *technology micro cluster* (tmc) comes into play: instead of simply counting the number of citations of patent A and calculating a quality score based on that number, we derive a tmc formed by *y* patents describing the technology that is closest to the one codified in patent A. We then use this tmc's average technological quality and adjust it for the patents that are assigned to the company of patent A. In our case, the quality of the tmc is derived based on the citation frequency of the patents in the tmc, which is then assigned to patent A (not necessarily to the other patents in the tmc, because their tmc can differ). However, as stated above, alternative quality definitions are possible as well. We identify the closest patents to patent A and define its tmc based on machine learning process, because machine learning approaches are particularly well suited to calculate the textual similarity between patent texts (see e.g., Risch et al., 2020; Seokkyu et al., 2022). Furthermore, it has been shown that text can be a powerful predictor to evaluate a patent's quality, making text-based machine learning techniques a strongly emerging approach in the literature (see e.g., Chung and Sohn, 2020 or Liu et al., 2020). For our purpose, we follow this emerging trend and use a method based on Sentence-BERT (see Reimers and Gurevych, 2019): Sentence-BERT is a transformer-based approach (see Vaswani et al., 2017; Devlin et al., 2018) that can be effectively applied to estimate textual similarities (Giancarlo, 2015). The corresponding pairwise similarity scores, sc, of a patent A with all other patents can then be ranked to find the most closely related patents for patent A.

Following this logic, we calculate the tmc for patent A (owned by firm *f* ) based on the set of all patents described by *N*_α*, i*_, *N*_α*, i-*1_, *N*_α*, i-*2_… *N*_α*, i-y*_, plus patent A itself, adjusted by the patents publications of firm *fN*__*f*_, α*, i*_, *N*__*f*_, α*, i-*1_, *N*__*f*_, α*, i-*2_… *N*__*f*_, α*, i-y*_ for the index α and the period *i, i-*1*, i-*2 …*i-y* yielding the set *N*_tmc_ (patent A):


(1) $$\begin{array}{l} N_{tmb}(patent\;A)\;=\;\max_{sc,y}\left(\;{⋃}_{j=1}^{y}N_{\alpha ,1-j}\backslash {⋃}_{k=1}^{y}N_{f,\alpha ,1-k}\right)\\ \;+patent\;A \end{array}$$

max_sc,y_ means the selection of the *y* patents with highest similarity score owned by other firms than firm *f*. The quality of patent A *q* (patent A) is then defined as the mean value of the quality values (indicted by a bar over the *q*) of the *N*_*tmb*_(patent A) patents:


(2) $$\begin{array}{l} q\left(patent\;A\right)=\frac{1}{y+1}\left(\sum_{i=1}^{y}{\bar{q}}_{i}+\bar{q}\left(patent\;A\right)\right) \end{array}$$

At this point it should be noted that the set of patents *M*_α*, i*_, *M*_α*, i-*1_, *M*_α*, i-*2_… *M*_α*, i-n*_ that forms the index alpha does not necessarily have to match the set of patents *N*_α*, i*_, *N*_α*, i-*1_, *N*_α*, i-*2_… *N*_α*, i-y*_ that is used to calculate the tmc. However, it is advisable to select patents for which a quality can be derived. For example, if the citation frequency is the method of choice, the selected patents *N*_α*, i*_, *N*_α*, i-*1_, *N*_α*, i-*2_… *N*_α*, i-y*_ should be of a certain age to have received any citations at all.

Having outlined our chosen approach to approximate a patent's technological quality, we can now describe in more detail how we operationalize our 4 measures that define the DynaPTI indicator.

### Sub-measure 1a: dynamic technological quality

*M*__*f*_, α, *i*_ is the number of patents published in year *i* corresponding to firm *f* in the index α. The mean value of the technological quality *q* of these patents yields *Q*_*f*, α, *i*_ = *mean*(*q*). Correspondingly, the technological quality of firm *f* assigned to the patents *M*__*f*_, α*, i-*1_, *M*__*f*_, α*, i-*2_… *M*__*f*_, α*, i-n*_ is given by *Q*__*f*_, α*, i-*1_, *Q*__*f*_, α*, i-*2_… *Q*__*f*_, α*, i-n*_. To calculate the technological quality change rate (sub-measure 1a), we first calculate the growth, i.e., the slope β(*Q*_*f*__, α*, i*_, *Q*__*f*_, α*, i-*1_, *Q*__*f*_, α*, i-*2_… *Q*__*f*_, α*, i-n*_) = β(*Q*__*f*_, α*, i, n*_) of the linear regression line fitting the values *Q*__*f*_, α*, i*_, *Q*__*f*_, α*, i-*1_, *Q*__*f*_, α*, i-*2_… *Q*__*f*_, α*, i-n*_. The slope of the regression line is calculated based on the least squares method according to


(3) $$\beta \left(Q_{f,\alpha ,i,n}\right)=\frac{\sum_{j=i-n}^{i}\left(j-{\bar{j}}_{i,n}\right)\cdot \left(Q_{\text{f},\alpha ,j}-{\bar{Q}}_{f,\alpha ,i,n}\right)}{\sum_{j=i-n}^{i}{\left(j-{\bar{j}}_{i,n}\right)}^{2}},$$

with the arithmetic mean value of the quality


(4) $$\begin{array}{l} {\bar{Q}}_{f,\alpha ,i,n}=\frac{1}{n+1}\sum_{j=i-n}^{i}Q_{f,\alpha ,j} \end{array}$$

and the arithmetic mean value of the due date years


(5) $$\begin{array}{l} {\bar{j}}_{i,n}=\frac{1}{n+1}\sum_{j=i-n}^{i}j=i-\frac{n}{2}. \end{array}$$

β(*Q*_*f*, α, *i, n*_) represents the absolute growth and hence the absolute change of the technological quality of firm *f* assigned to the index α with the patent portfolios *M*__*f*_, α*, i*_, *M*__*f*_, α*, i-*1_, *M*__*f*_, α*, i-*2_… *M*__*f*_, α*, i-n*_. To calculate the relative growth γ(*Q*_*f*, α, *i, n*_) of the technological quality, we divide the absolute growth by the arithmetic mean value according to


(6) $$\begin{array}{l} \gamma \left(Q_{f,\alpha ,i,n}\right)=\frac{\beta \left(Q_{f,\alpha ,i,n}\right)}{{\bar{Q}}_{f,\alpha ,i,n}}\\ \;=\left(n+1\right)\frac{\sum_{j=i-n}^{i}\left(j-{\bar{j}}_{i,n}\right)\cdot \left(Q_{f,\alpha ,j}-{\bar{Q}}_{f,\alpha ,i,n}\right)}{\sum_{l=i-n}^{i}Q_{f,\alpha ,l}\sum_{j=i-n}^{i}{\left(j-{\bar{j}}_{i,n}\right)}^{2}}. \end{array}$$

To relate firm *f* 's relative growth of the technological quality of its patent portfolios *M*_f,α*,i*_, *M*_f,α*,i-*1_, *M*_f,α*,i-*2_… *M*_f,α*,i-n*_ to the index α consisting of *c* firms, we apply the method of the statistical standard score. For this purpose, γ(*Q*_**α**, *i, n*_), the mean value of the relative growth of the technological quality of all firms forming the index α, is calculated according to


(7) $$\begin{array}{l} \gamma \left(Q_{\alpha ,i,n}\right)=\frac{1}{c}\cdot \sum_{f=1}^{c}\gamma \left(Q_{f,\alpha ,i,n}\right)=\frac{1}{c}\cdot \sum_{f=1}^{c}\frac{\beta \left(Q_{f,\alpha ,i,n}\right)}{{\bar{Q}}_{f,\alpha ,i,n}}\\ \;=\frac{n+1}{c}\cdot \sum_{f=1}^{c}\frac{\sum_{j=i-n}^{i}\left(j-{\bar{j}}_{i,n}\right)\cdot \left(Q_{f,\alpha ,j}-{\bar{Q}}_{f,\alpha ,i}\right)}{\sum_{l=i-n}^{i}Q_{f,\alpha ,l}\cdot \sum_{j=i-n}^{i}{\left(j-{\bar{j}}_{i,n}\right)}^{2}}. \end{array}$$

The standard deviation *d*_*Q*,α,*i,n*_ is given by


(8) $$\begin{array}{l} d_{Q,\alpha ,i,n}=\sqrt{\frac{1}{c}\sum_{f=1}^{c}{\left(\gamma \left(Q_{f,\alpha ,i,n}\right)-\gamma \left(Q_{\alpha ,i,n}\right)\right)}^{2}}. \end{array}$$

We can then use these calculations to derive an index-based change rate for the technological quality of firm *f* in the time period *i, i-*1*, i-*2…*i-n* according to the standard score


(9) $$\begin{array}{l} \delta_{Q,f,\alpha ,i,n}=\frac{\gamma \left(Q_{f,\alpha ,i,n}\right)-\gamma \left(Q_{\alpha ,i,n}\right)}{d_{Q,\alpha ,i,n}}. \end{array}$$

### Sub-measure 1b: dynamic technological activity

The technological activity change rate related to the index α is calculated in the same way as the technological quality change rate. The technological activity of firm *f* assigned to patents *M*_*f*, α, *i*_, *M*__*f*_, α*, i-*1_, … *M*__*f*_, α*, i-n*_ is given by *M*__*f*_,α, *i*_, *M*__*f*_,α*, i-1*_…*M*__*f*_,α*, i-n*_ itself: The number of patents published in the corresponding year. To calculate the change rate of the activity (sub-measure 1b), we first calculate again the growth, i.e., slope β(*M*__*f*_,α*, i*_, *M*__*f*_,α*, i-*1_, *M*__*f*_,α*, i-*2_…*M*__*f*_, α*, i-n*_) = β(*M*__*f*_,α*, i, n*_) of the linear regression line fitting the values *M*__*f*_,α*, i*_, *M*__*f*_,α*, i-*1_, *M*__*f*_,α*, i-*2_…*M*__*f*_, α*, i-n*_. The slope of the regression line is calculated based on the least squares method according to


(10) $$\begin{array}{l} \beta \left(M_{f,\alpha ,i,n}\right)=\frac{\sum_{j=i-n}^{i}\left(j-{\bar{j}}_{i,n}\right)\cdot \left(M_{f,\alpha ,j}-{\bar{M}}_{f,\alpha ,i,n}\right)}{\sum_{j=i-n}^{i}{\left(j-{\bar{j}}_{i,n}\right)}^{2}}, \end{array}$$

with the activity's arithmetic mean value


(11) $$\begin{array}{l} {\bar{M}}_{f,\alpha ,i,n}=\frac{1}{n+1}\sum_{j=i-n}^{i}M_{f,\alpha ,j} \end{array}$$

and ${\bar{j}}_{i,n}\;$as the arithmetic mean value of the due date years (equation 5). β(*M*_*f*, α, *i, n*_) represents the absolute growth and the absolute change of the activity of firm *f* assigned to the index α with patents *M*__*f*_,α, *i*_, *M*__*f*_,α*, i-1*_…*M*__*f*_,α*, i-n*_.

The information value of absolute changes must be considered as rather limited since this sub-measure can be biased in favor of firms with higher patenting activity. Hence, it is expected that firms with large patent portfolios tend to have larger absolute growth rates when referring to the change of the absolute number of active patent families. To account for this distorting size effect, the activity's relative growth γ(*M*_*f*, α, *i, n*_) is calculated by dividing the absolute growth by the arithmetic mean value according to


(12) $$\begin{array}{l} \gamma \left(M_{f,\alpha ,i,n}\right)=\frac{\beta \left(M_{f,\alpha ,i,n}\right)}{{\bar{M}}_{f,\alpha ,i,n}}\\ \;=\left(n+1\right)\frac{\sum_{j=i-n}^{i}\left(j-{\bar{j}}_{i,n}\right)\cdot \left(M_{f,\alpha ,j}-{\bar{M}}_{f,\alpha ,i,n}\right)}{\sum_{l=i-n}^{i}M_{f,\alpha ,l}\sum_{j=i-n}^{i}{\left(j-{\bar{j}}_{i,n}\right)}^{2}}. \end{array}$$

To relate the relative growth regarding the technological activity determined by the patent portfolio *M*__*f*_,α, *i*_, *M*__*f*_,α*, i-1*_…*M*__*f*_,α*, i-n*_ of firm *f* to the index α consisting of *c* firms, we again apply the method of the statistical standard score. For this purpose, we calculate γ(*M*_α, *i, n*_), the mean value of the relative growth of the technological activity of all firms forming the index α according to


(13) $$\begin{array}{l} \gamma \left(M_{\alpha ,i,n}\right)=\frac{1}{c}\cdot \sum_{f=1}^{c}\gamma \left(M_{f,\alpha ,i,n}\right)=\frac{1}{c}\cdot \sum_{f=1}^{c}\frac{\beta \left(M_{f,\alpha ,i,n}\right)}{{\bar{M}}_{f,\alpha ,i,n}}\\ \;=\frac{n+1}{c}\cdot \sum_{f=1}^{c}\frac{\sum_{j=i-n}^{i}\left(j-{\bar{j}}_{i,n}\right)\cdot \left(M_{f,\alpha ,j}-{\bar{M}}_{f,\alpha ,i}\right)}{\sum_{l=i-n}^{i}M_{f,\alpha ,l}\cdot \sum_{j=i-n}^{i}{\left(j-{\bar{j}}_{i,n}\right)}^{2}}. \end{array}$$

The corresponding standard deviation *d*_*M*,α,*i,n*_ is given by


(14) $$\begin{array}{l} d_{M,\alpha ,i,n}=\sqrt{\frac{1}{c}\sum_{f=1}^{c}{\left(\gamma \left(M_{f,\alpha ,i,n}\right)-\gamma \left(M_{\alpha ,i,n}\right)\right)}^{2}}. \end{array}$$

Equivalent to the calculations for the technological quality, the index-based change rate of the technological activity for firm *f* in the time duration *i, i-*1*, i-*2…*i-n* is then given by


(15) $$\begin{array}{l} \delta_{M,f,\alpha ,i,n}=\frac{\gamma \left(M_{f,\alpha ,i,n}\right)-\gamma \left(M_{\alpha ,i,n}\right)}{d_{M,\alpha ,i,n}}. \end{array}$$

### Sub-measure 2a: average technological quality

To consider the mean value of the technological quality (sub-measure 2a) in a consistent manner with the change rates of the quality and activity, the mean value of the quality (equation 4) over the time duration i, i-1, i-2…i-n for all firms *f* forming the index α is calculated according to


(16) $$\begin{array}{l} {\bar{Q}}_{\alpha ,i,n}\;=\frac{1}{c}\cdot \sum_{f=1}^{c}{\bar{Q}}_{f,\alpha ,i,n}=\frac{1}{c}\cdot \frac{1}{n+1}\cdot \sum_{f=1}^{c}\sum_{j=i-n}^{i}Q_{f,\alpha ,j,n}. \end{array}$$

The standard deviation $d_{\bar{Q},\alpha ,i,n}$ of the mean quality representing the index α is then given by


(17) $$\begin{array}{l} d_{\bar{Q},\alpha ,i,n}=\sqrt{\frac{1}{c}\sum_{f=1}^{c}{\left({\bar{Q}}_{f,\alpha ,i,n}-{\bar{Q}}_{\alpha ,i,n}\right)}^{2}}. \end{array}$$

The formulas above yield the index-based mean value of the technological quality of firm *f* in the time period *i, i-*1*, i-*2…*i-n* according to the standard score


(18) $$\begin{array}{l} \delta_{\bar{Q},f,\alpha ,i,n}=\frac{{\bar{Q}}_{f,\alpha ,i,n}-{\bar{Q}}_{\alpha ,i,n}}{d_{\bar{Q},\alpha ,i,n}}. \end{array}$$

### Sub-measure 2b: average technological activity

The average value of the technological activity (sub-measure 2b) is derived equivalently to the corresponding calculations for the technological quality. The mean value of the technological activity (equation 11) over the time duration *i, i*-1, *i*-2…*i-m* for all firms *f* is calculated according to


(19) $$\begin{array}{l} {\bar{M}}_{\alpha ,i,m}\;=\frac{1}{c}\cdot \sum_{f=1}^{c}{\bar{M}}_{f,\alpha ,i,m}\\ \;=\frac{1}{c}\cdot \frac{1}{m+1}\cdot \sum_{f=1}^{c}\sum_{j=i-m}^{i}M_{f,\alpha ,j,m}. \end{array}$$

And the standard deviation $d_{\bar{M},\alpha ,i,n}$ of the mean activity representing the index α is given by


(20) $$\begin{array}{l} d_{\bar{M},\alpha ,i,m}=\sqrt{\frac{1}{c}\sum_{f=1}^{c}{\left({\bar{M}}_{f,\alpha ,i,m}-{\bar{M}}_{\alpha ,i,m}\right)}^{2}}. \end{array}$$

The formulas above yield the index-based mean value of the technological activity of firm *f* in the time period *i, i*-1, *i*-2…*i-m* according to the standard score


(21) $$\begin{array}{l} \delta_{\bar{M},f,\alpha ,i,m}=\frac{{\bar{M}}_{f,\alpha ,i,m}-{\bar{M}}_{\alpha ,i,m}}{d_{\bar{M,}\alpha ,i,m}}. \end{array}$$

Note that the mean value of the technological activity is given here for *m* instead of *n* years like the other sub-measures. Of course, *m* can be set to *n*, but in practice the current technological activity (e.g., *m* = 2) of a company is often considered instead of a longer time period, so in many cases *m*<*n*.

### Wrapping up: aggregation to DynaPTI

The final value of DynaPTI, δ_*f*, α, *i, n, m*_, is calculated by simply adding the four sub-measures up. That is, the index-based change rate of firm *f* 's technological quality, δ_*Q, f*, α, *i, n*_, the index-based change rate of its technological activity δ_*M, f*, α, *i, n*_, the index-based mean value of its technological quality, $\delta_{\bar{Q},f,\alpha ,I,n}$, and the index-based mean value of its technological activity, $\delta_{\bar{M},f,\alpha ,i,m}$, together determine the final value of our indicator.


(22) $$\begin{array}{l} \delta_{f,\alpha ,i,n,m}=\delta_{Q,f,\alpha ,i,n}+\delta_{M,f,\alpha ,i,n}+\delta_{\bar{Q},f,\alpha ,i,n}+\delta_{\bar{M},f,\alpha ,i,m} \end{array}$$

Due to the method of standard score, the mean value of δ_*f*, α, *i, n, m*_ *i*s 0:


(23) $$\begin{array}{l} \sum_{f=1}^{c}\delta_{f,\alpha ,i,n,m}=0 \end{array}$$

Firm *f* can achieve a high indicator value if it has (i) a high technological quality (ii) a high technological activity (iii) an increasing technological quality, (iv) an increasing technological activity. Firms with an indicator value, δ_*f*, α, *i,n,m*_ > 0, are above average according to the DynaPTI measure, whilst firms with δ_*f*, α, *i, n, m*_ < 0 are below average. We next provide an illustration of this framework to demonstrate its implementation and main benefits.

## An application to the wind energy sector

In what follows, we demonstrate an application of the DynaPTI framework to present how it can be implemented and operationalized. We perform this application exercise for firms that are active inventors in over the recent past the technology field of wind energy. Although the primary goal of this application is to present an example implementation and not an assessment of a technology field, this selection to firms active in wind energy allows us to highlight how our proposed indicator can be used for corporate intelligence in a technology field that is highly important for the green transformation.

In order to do this, the first step is to define an index α of firms from the technology field of wind energy and to extract their corresponding patents for a given time window. We identify such firms and their patents based on data and a definition of wind energy patents provided by EconSight AG.^11^ In addition, we retrieve the following measures to calibrate DynaPTI's 4 sub-indicators: the publication date for each patent, the total patent portfolio size for every firm, the portfolio share in wind energy for each owner, and the quality for each patent derived at the end of 2022. Note that as an approximation of patents' technological quality, we thus again use a definition developed by EconSight, which is based on a patent's received citations from today's perspective. Accordingly, a patent that is published around 2020 and thus assigned to 2020 based on our definitions, is rated with its quality score from 2022.^12^

We then set the following restrictions and use the following parameters to calibrate the framework: First, to keep our analysis concise, we require that all patents we use for any kind of calculation must be assigned to EconSight's wind energy technology field. Second, we want to focus on relevant players in this area. To ensure this, we restrict our selection of patents to those from firms that have published at least 5 patents in the time period 2020–2022, and have at least 1% of their patents in the field of wind energy. Third, we aim to focus on the very recent past to have an up-to date evaluation. Hence, we restrict the selection of patents to wind energy patents published in the 3 years' time period 2020–2022 for the calculation of our 4 sub-indicators. Instead, the set of patents for calculating the tmc is formed by all patents in the field of wind energy, which were published in the time period 2015–2018, and is not restricted to any set of firms. Again, with regard to the tmc, the number of patents to calculate the parameter *y* in equations (1) and (2) is set to *y* = 5. The DynaPTI for innovators in wind energy is then derived according to the specifications in the previous section and calculated using a Python script. The corresponding results can be seen in Tables 1, 2.

**Table 1:** Parameters for wind energy.

**Parameter**	**Value**
γ(*Q*_α, *i, n*_)	−0.04
γ(*M*_α, *i, n*_)	0.05
${\bar{Q}}_{\alpha ,i,n}$	2.93
${\bar{M}}_{\alpha ,i,m}$	42.5
*d*_*Q*, α, *i, n*_	0.18
*d*_*M*, α, *i, n*_	0.42
$d_{\bar{Q},\alpha ,i,n}$	0.47
$d_{\bar{M},\alpha ,i,m}$	113.5

**Table 2:** Firms ranked by DynaPTI with patent portfolio in wind energy.

**Current owners**	**(A) DynaPTI**	**(B) 1a Dynamic Quality**	**(C) 1b Dynamic Activity**	**(D) 2a Average Quality**	**(E) 2b Average Activity**	**(F) Specialization**	**(G) PAI**	**(H) WNDY Index Weight**
Siemens Energy AG	3.98	0.12	−0.23	−0.04	4.13	26.1	4,661	4.59%
Vestas Wind Systems AS	3.52	0.78	−0.50	0.02	3.22	90.0	5,379	13.89%
Vinci SA	2.22	0.08	1.93	0.53	−0.32	4.2	24	-
ThyssenKrupp AG	1.31	0.76	0.63	0.18	−0.25	1.3	133	-
Holcim Ltd	0.84	−0.76	−0.13	2.08	−0.35	2.7	74	-
Senvion SA	0.38	0.75	−0.80	0.40	0.03	96.6	77	-
NTN Corp	0.24	0.08	0.01	0.38	−0.24	2.7	223	-
SKF AB	0.12	−0.14	−0.13	0.66	−0.28	3.3	157	-
Equinor ASA	0.03	−0.04	0.19	0.19	−0.31	4.1	143	-
Moog Inc	−0.11	0.23	−0.13	0.16	−0.37	9.8	35	-
Siemens AG	−0.60	−0.35	−0.56	0.47	−0.15	3.2	195	-
Nabtesco Corp	−0.66	−0.03	−0.78	0.46	−0.31	3.4	61	-
Nordex SE	−0.71	0.23	−1.09	0.28	−0.13	93.8	377	2.66%
Orsted AS	−1.04	−0.48	−0.41	0.12	−0.27	36.7	96	11.94%
RWE AG	−1.53	0.59	−1.38	−0.59	−0.15	22.9	119	-
Mitsubishi Heavy Industries Ltd	−1.56	−0.67	−0.52	−0.22	−0.15	5.4	12,55	-
Saipem SpA	−2.60	−1.41	0.59	−1.44	−0.34	3.2	43	-
EOn SE	−2.87	−0.87	−1.64	−0.01	−0.35	12.1	4	-

In Table 1 we present the parameters calculated according to equations (7, 8, 12, 13, 16, 17, 19 and 20). The first 4 values represent the respective index mean values for wind energy, the last 4 values the corresponding standard deviation. The results for the DynaPTI calculations are given in Table 2. According to equation (22), the DynaPTI is simply the sum of its 4 sub-indicators. To provide an indication for the drivers of the overall results, we have also separately specified the individual sub-indicators 1a, 1b, 2a, and 2b in the Table 2. These values can be seen in columns (B) to (E). Column (F) highlights every firm's degree of specialization in wind energy technologies, depicted by the share of their overall patents attributed to wind energy. Finally, columns (G) and (H) present similar information from alternative sources, which we can use to contrast our findings: Column (G) states firm-ratings based on the Patent Asset Index from PatentSight, which is a static patent-based technology indicator (Ernst and Omland, 2011). Column (H), in turn, highlights firms' portfolio weight in the popular *Global X Wind Energy ETF* (if they are included in the fund).

According to our results, the two companies Siemens Energy AG and Vestas Wind Systems AS show particularly high DynaPTI values. Both companies have a very high sub-measure 2b and thus a particularly high patent activity in the technology field wind energy. Compared to the other companies in the index, Vestas Wind Systems AS also has the highest sub-measure 1a and thus the highest dynamic in the development of patent quality.

Another interesting case is Vinci SA, which has a particularly high sub-measure 1b. This indicates that its patent activity has increased significantly in the last 3 years compared to the other companies. Moreover, the company Holcim Ltd has a particularly high sub-measure 2a and thus a particularly high average patent quality compared to the other companies. This means that the companies Holcim Ltd and Vinci SA are also rated very highly in the DynaPTI even though they only have a relatively small proportion of their overall patents in the wind energy sector (see column F).

Turning to a comparison with alternative measures, let us first focus on column (G), where we have included the Patent Asset Index (PAI) scores from PatentSight, which is an established patent-based indicator for corporate intelligence that although lacks a dynamic component.^13^ We can immediately see a strong deviation between this static indicator and our proposed dynamic method: While the PAI is particularly (but not exclusively) determined by large patent portfolios, DynaPTI instead places a strong focus on the dynamics in the development of the patent portfolios. Therefore, relatively large players such as Nordex SE are rated quite highly by the PAI, but not by DynaPTI. Incorporating a dynamic perspective, DynaPTI assigns a rather low value to Nordex's dynamic activity, suggesting that its recent activity has been below average relative to the index.^14^ This example clearly demonstrates a key strength of our proposed framework.

A second main advantage of our indicator can be seen from column (H), where we report companies' portfolio weights in the *Global X Wind Energy ETF* if they are present in the fund. The two top-performing companies according to DynaPTI are also included in the ETF and have relatively high portfolio weights. However, several green innovation over-performers, such as Vinci, ThyssenKrupp or Holcim are not reflected in this ETF. This may be due to their relatively low specialization in wind energy. Yet, despite their relatively low overall specialization our dynamic patent technology indicator suggests that they may be important innovators in wind energy and could make significant new contributions to this technology field. Thus, our approach can possibly offer investors a data-driven framework to identify companies that may be neglected due to their size or specialization. Conversely, it might also be a tool to detect potential greenwashing from companies pretending to innovate in an ESG-related field but not showing significant performance based on our framework.

## 5. Discussion and conclusion

Patent data has become an established source of information for both scientific research and corporate intelligence. However, most patent-based indicators fail to consider firm-level dynamics regarding technological quality and technological activity. Especially in rather dynamic technological areas that are particularly relevant for the green transition, this limitation can distort company assessments that are based on existing patent-based measures. In this paper, we have developed a framework to build a novel indicator, which we call DynaPTI, that tackles this particular shortcoming. Its main strength is that it provides researchers and practitioners a tool that is more reliable for up-to-date assessments about firm-level innovation activities.

Our proposed approach is complementary to prior work and is based on three main building blocks: First, it focuses on index-based comparisons to mitigate distorting effects such as the technological area. Second, it uses a transformer-based approach to leverage textual information contained in patents. This procedure allows to approximate the technological quality of firms' innovation pipelines and to correct for distorting factors related to the patent examination process. Third, the indicator consists of four different sub-measures that capture technological quality and activity, as well as their corresponding dynamics over a specific time window. We operationalize these sub-measures using patent citations and patent applications as approximations for the technological quality and activity of firms, respectively. Note however that our proposed indicator is relatively generic and can be flexibly specified. Researchers and practitioners could thus easily adapt its empirical ingredients as long as there is corresponding temporal data available. For example, one may use patent claims instead of patent citations as a technological quality proxy.

Yet, the main advantage of our proposed indicator is that it incorporates a dynamic perspective. This seems particularly useful for investigating companies in technological areas that are characterized by rapid changes, which is the case, for example, for several technology domains that are highly relevant for a successful green transition. We demonstrate this by providing an application of our framework to the wind energy sector. In doing so, we assess the strength of companies' innovation pipelines that are active in this domain based on different measures, including our own. Taken together, the results from this application exercise highlight the following: DynaPTI may be viewed as a tool that can uncover recently emerging innovation-overperformers in a particular technological field and is less biased in favor of large incumbents compared to existing patent-based indicators.

We demonstrate that our framework can detect significant innovators that tend to be neglected or are not considered at all by a popular ETF focused on wind energy. Furthermore, we show that DynaPTI can uncover emerging companies in this field that are albeit rather modestly rated by an established but static patent indicator, such as the *Patent Asset Index*. This is because DynaPTI puts less to no emphasis on innovations from the relatively distant past compared to static patent-based innovation indicators (e.g., Grimaldi et al., 2015 or Ernst and Omland, 2011). It thus provides a more up-to-date assessment of companies' innovative pipelines. This is a particularly compelling feature when one's aim is to evaluate a company's *recent* inventive activities.

However, for different purposes, static indicators may have certain advantages. For example, the aggregate strength of a certain company's overall patent portfolio might be better assessed using static indicators since they take all patented inventions of this company equally into account. Hence, we view our proposed framework primarily as a complement to existing methods. The model also has some further limitations that must be taken into account in practice. While our application clearly illustrates the potential usefulness of dynamic patent indicators, it also remains limited to one specific technology domain and a certain time window due to the scope of this paper. Furthermore, the model parameters also require further emphasis: There can be different perspectives regarding the definition of some key parameters: For example, which period should be considered, which time frame should be chosen for calculating the average patenting activity, how many patents should be considered in the technology micro cluster (tmc), how strongly is the calculation of the tmc influenced by the selected machine learning model. These parameter choices may depend on the underlying technology or the selected index: the faster a technology develops, the shorter the period should be defined. The period can also depend on the context in which the relevant index calculations are carried out: longer periods may have to be chosen for political decisions, i.e., for decisions at the economic management level. Ultimately, one must also consider that for patents with a completely new technological approach, the tmc calculation may lead to nonsensical outcomes, since such a patent would not have any semantically close counterparts.

Hence, future research in the area of patent-based technology indicators may primarily focus on evaluating our measure's performance and those of other dynamic approaches in alternative settings. Another direction for future research would be to examine the parameter space of the model and, if necessary, identify meaningful values depending on the scenario. Furthermore, since our proposed framework is rather generic, it would be interesting to specify and calibrate DynaPTI based on alternative parameters (e.g., the length of the time window) and data (e.g., patent claims instead of patent citations), and subsequently evaluate corresponding results. In principle, DynaPTI can even be expanded beyond patent data. Due to its index-based calculation and the method of the statistical standard score, parameters can be omitted or added at will. For example, it would be possible to include financial key figures such as sales and profits. With this regard, DynaPTI could also be interpreted and tested as an extension tool for classical financial analyses. Taken together, we believe that all of these open issues are interesting and important avenues for future research to improve patent-based indicators for scientific research and corporate intelligence.

## Data availability statement

The data analyzed in this study is subject to the following licenses/restrictions: data is proprietary. Requests to access these datasets should be directed to michael.freunek@econsight.ch.

## Author contributions

MF: conceptual idea, methodological approach and calculation, empirical estimation, and conclusions. MN: literature, motivation, methodological evaluation, empirical evaluation, and conclusions. Both authors contributed to the article and approved the submitted version.

## Footnotes

^1^For some of the potentials and the importance of green investments for the green transition, see for example, Falcone (2020), Polzin et al. (2019) or Eyraud et al. (2013) for some recent contributions. A more general framework regarding directed technical change towards the development of green technologies is provided by Acemoglu et al. (2012) or Popp (2002).

^2^Naturally, the use of patent statistics also has its challenges, which are discussed extensively in the literature. See, for example, Lerner and Seru (2022), Fontana et al. (2013) or Griliches (1998).

^3^Note that our framework can be easily enriched or adapted with alternative quality measures (e.g., the number of patent claims). Since patent citations is one of the most established measures in the literature (see e.g., Nagaoka et al., 2010), we use it in this paper to demonstrate our methodological concept.

^4^See, for example, Arts et al. (2021), Lee et al. (2018), Grimaldi et al. (2015), Thoma (2014), Gerken and Moehrle (2012) or Ernst and Omland (2011) for some recent contributions. An overview of different approaches and applications can be found in Dziallas and Blind (2019), or Nagaoka et al. (2010).

^5^See, for example, Harhoff et al. (1999) or Hall et al. (2005) for some seminal contributions regarding the use of patent citations. An overview of best practices can be found in Jaffe and de Rassenfosse (2019).

^6^A selection of studies using patent data to focus on innovation at different aggregation levels are, for example, Yamashita (2021) for a country-level analysis, Ernst and Omland (2011) for the company-level, or Carlino et al. (2007) regarding regional innovation hubs.

^7^The idea of a patent family is to group patents that jointly protect the same invention. For more detailed information on this well-established concept, see, for example, Dernis and Khan (2004).

^8^In different contexts, similar approaches have been applied by Hain et al. (2022) or Arts et al. (2021).

^9^An example could be that patent examiners are constantly asking for the citation of the same, well-known patents. For an overview regarding the treatment of patent citations see e.g., Jaffe and de Rassenfosse (2019).

^10^Note that the list of exemplary indices is non-exhaustive. Indices to which firms patent portfolios are compared can be freely defined, either as existing indices for which examples were mentioned above or by defining own sets of firms to which the firm is compared.

^11^This technology field includes technologies like wind turbines, rotor blades and switch gears.

^12^Alternatively, you can also work with the patent quality indicators for each year, which applied to that year and not from today's perspective.

^13^The Patent Asset Index is the sum of the competitive impact of all patent families of a company (here in the index α).

^14^It should be noted once again that the average value of the DynaPTI and of all sub-indicators 1a to 2b is 0 in each case. Values greater than 0 are therefore above average, values <0 are below average. Further companies such as EOn SE and Mitsubishi Heavy Industries Ltd are therefore also below average performers in all 4 sub-measure and are thus positioned very low in the ranking.
